# Wavelength-Sensitive Superconducting Single-Photon Detectors on Thin Film Lithium Niobate Waveguides

**DOI:** 10.1021/acs.nanolett.3c02324

**Published:** 2023-10-23

**Authors:** Alessandro Prencipe, Samuel Gyger, Mohammad Amin Baghban, Julien Zichi, Katharina D. Zeuner, Thomas Lettner, Lucas Schweickert, Stephan Steinhauer, Ali W. Elshaari, Katia Gallo, Val Zwiller

**Affiliations:** Department of Applied Physics, KTH Royal Institute of Technology, Roslagstullsbacken 21, Stockholm SE-106 91, Sweden

**Keywords:** thin film lithium niobate, superconducting nanowire single-photon detector, on-chip wavelength meter, on-chip single-photon detector

## Abstract

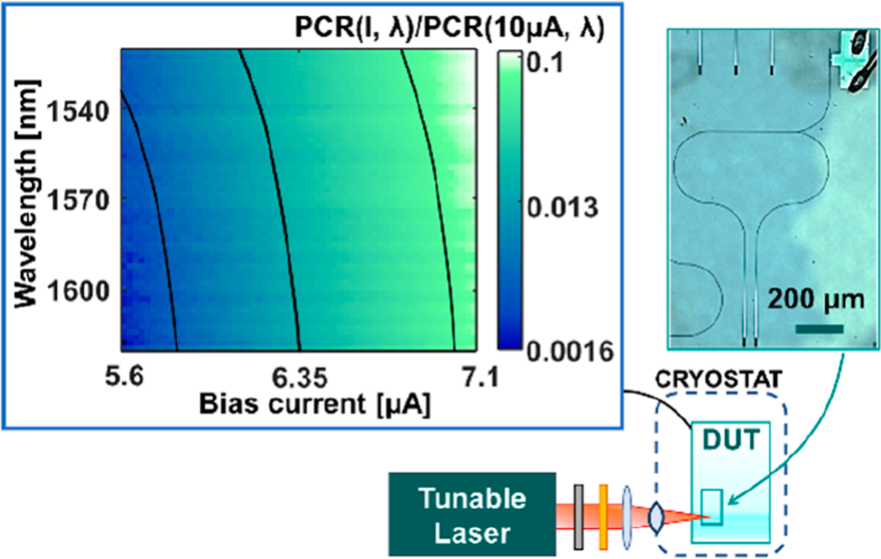

Lithium niobate, because of its nonlinear and electro-optical properties, is one of the materials of choice for photonic applications. The development of nanostructuring capabilities of thin film lithium niobate (TFLN) permits fabrication of small footprint, low-loss optical circuits. With the recent implementation of on-chip single-photon detectors, this architecture is among the most promising for realizing on-chip quantum optics experiments. In this Letter, we report on the implementation of superconducting nanowire single-photon detectors (SNSPDs) based on NbTiN on 300 nm thick TFLN ridge nano-waveguides. We demonstrate a waveguide-integrated wavelength meter based on the photon energy dependence of the superconducting detectors. The device operates at the telecom C- and L-bands and has a footprint smaller than 300 × 180 μm^2^ and critical currents between ∼12 and ∼14 μA, which ensures operation with minimum heat dissipation. Our results hold promise for future densely packed on-chip wavelength-multiplexed quantum communication systems.

Photonic integrated circuits (PICs) offer an important advantage compared to tabletop optics in terms of scalability, footprint, stability, and low power consumption.^1−4^ Electro-optical materials are particularly attractive as a material platform for PICs because of the opportunity of implementing on-chip high-speed reconfigurable optical systems necessary for a number of applications in optical communication,^5^ computation,^6^ and sensing.^7^ Lithium niobate has been for decades the material of choice for integrated photonics, not only because of its electro-optical properties: its noncentrosymmetric structure makes it suited also for three-wave mixing processes, needed for efficient on-chip nonlinear optics. Since ultralow-loss PICs have been demonstrated in thin film lithium niobate (TFLN),^8^ the interesting properties of this material have been leveraged also in nanophotonic circuits, demonstrating disruptive results in quantum photonics,^9^ nonlinear optics,^10^ and ultrafast modulation of light on a chip.^5^ In recent years, on-chip detection has also been proven on TFLN optical circuits, taking advantage both of silicon detectors^11^ and superconducting nanowire single-photon detectors (SNSPDs).^9,12,13^ SNSPDs offer very competitive performance in terms of footprint, efficiency, time resolution, and noise when compared to single-photon detectors based on other technologies.^14^ Waveguide-integrated SNSPDs can add to these performances better coupling efficiency to the waveguide optical mode, higher maximum count rates, short latency, and an even smaller footprint.^15^ The scalable integration of efficient single-photon detectors on TFLN holds promises for the waveguide integration of complex quantum optics experiments, in a similar fashion to what has recently been done in other materials^16^ and on traditional titanium-diffused optical circuits in bulk lithium niobate,^17^ a technological platform where the use of periodically poled waveguides for quantum applications in both single-photon^17^ and continuous variable quantum regimes^18^ is already mature.

In this work, we demonstrate hairpin SNSPDs integration on single-mode *x*-cut lithium niobate nano-waveguides to utilize them as wavelength meter in the wavelength range between 1520 and 1630 nm. The wavemeter functionality of SNPDs has been previously shown with free-space excitation from multimode fibers.^19^ However, the excellent performances achieved with waveguide-integrated SNSPDs,^9,12,13^ and their growing relevance and applications in the context of photonic integrated circuits, motivate the interest in exploiting their spectral-resolving capabilities in fully integrated on-chip formats. SNSPDs show an increasing sensitivity with higher bias current until they finally stop operating due to high dark counts and reach their critical current. The detected count rate as a function of the bias current for a constant photon flux follows a sigmoid curve and saturates once all absorbed photons are detected. The responsivity of the detector is dependent on the photon energy,^19^ which allows to measure the wavelength by sampling different points on the count-rate curve. Such dependence is common to SNSPDs and has been justified, within the framework of the hot spot model, considering an increase of the size of the resistive region with the photon energy: the hot spot diameter *D* scales inversely with the wavelength λ of the detected photon, as *D* ∝ .^20^ This phenomenon leads to a spectral dependence of the device latency^21^ and to an energy detection threshold for SNSPDs,^22^ setting a limit to the useful range of these detectors. However, it can also be used to realize new on-chip, wavelength-sensitive complex functionalities. We explore this opportunity by performing a simple analysis of the light signal measured by waveguide-integrated SNSPDs at different bias currents and prove that this allows us to exploit our detectors as on-chip wavelength meters for the telecom C- and L-bands.

The devices are fabricated starting from a commercial wafer of 300 nm thick *x*-cut TFLN from NANOLN. The fabrication flow is presented in Figure 1.

**Figure 1:**
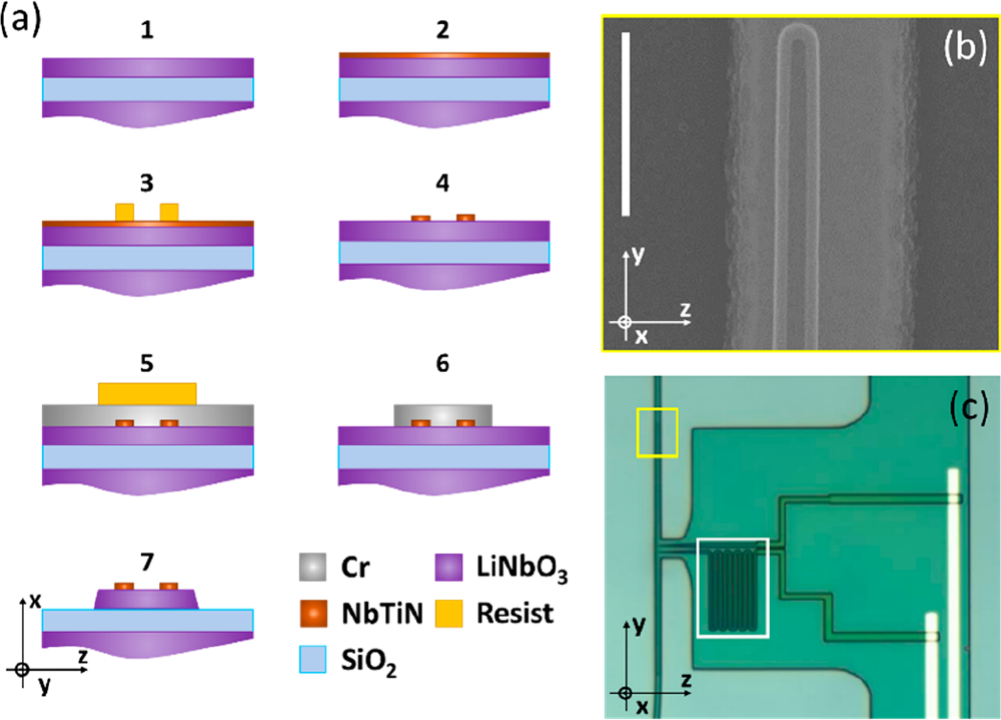
(a) Fabrication workflow. A first e-beam lithography step defines the superconducting circuit on a NbTiN film sputtered on a 300 nm thick LiNbO_3_ layer. The NbTiN film is dry etched before evaporating a Cr hard mask. A second e-beam lithography followed by Cl_2_-based dry etching serves to define the Cr mask that protects the TFLN during Ar^+^ milling. (b) SEM image of a SNSPD on top of a waveguide, taken as a zoomed-in image on the yellow box in (c). The scale bar is 1 μm. (c) Microscope view of the device, showing also the on-chip inductance (white box). *x*, *y*, and *z*: TFLN crystal axes.

First, a 8 nm thick NbTiN layer is deposited on the TFLN by reactive cosputtering from separate Nb (120 W, DC) and Ti (240 W, RF) targets at room temperature in nitrogen and argon atmospheres.^23^ Cr/Au markers defined by lift-off (AR6200.18, 80 nm thick) are used to align a first electron beam lithography (Raith, Voyager, 50 kV) of 300 nm thick ma-N2400 negative tone resist followed by CF_4_/O_2_ reactive ion etching (Oxford, Plasmalab 100). In this way, NbTiN nanowires are defined on known positions on the chip. A 300 nm thick layer of Cr is evaporated on the sample, and 500 nm of ma-N2400 resist is used to pattern in a single-pass electron beam lithography (50 kV) both optical waveguides and grating couplers properly placed with respect to the detectors. Cl_2_-based reactive ion etching is used to transfer the resist pattern to the Cr layer, which acts as a hard mask during Ar^+^ milling of the lithium niobate.^24^ Finally, the Cr mask is stripped by wet etching (Sigma-Aldrich, Cr etchant standard). The chemical composition of the NbTiN film grants good chemical stability to the detectors during optical circuit fabrication. The uncladded waveguides are single mode and feature a thickness of 300 nm and a width of ∼700 nm. The optical loss, evaluated by means of test structures, is ∼5 dB/cm. Waveguides and detectors are aligned along the *y*-axis of lithium niobate, as visible in Figure 1. The nanowire width is 65 nm. The two NbTiN stripes are separated by 100 nm. The minimum footprint of one device is limited by the bonding pads used to contact the SNSPD. In our case it amounts to ∼300 × 180 μm^2^ but could be further reduced with smaller pads, and the footprint of the detector itself is comparable to the one of a 180 μm long waveguide.

Before performing waveguide coupled measurements, the detectors were precharacterized at 850, 1310, and 1550 nm in a flood illumination setup at 2.5 K. The devices were biased using a commercial SNSPD driver (Single Quantum EOS) with integrated counter. The detector response can be seen in Figure 2a, where the wavelength dependence is apparent.

**Figure 2:**
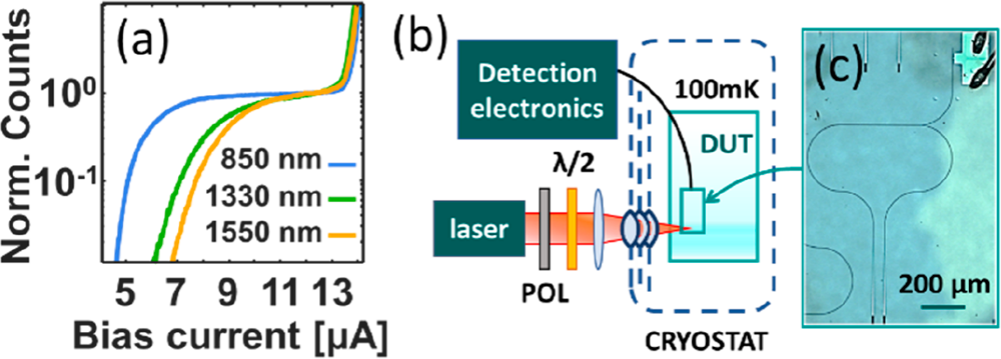
(a) Normalized photon count rate as a function of the bias current for flood illumination at 850, 1330, and 1550 nm. The critical current for this device amounts to *I*_C_ ∼ 14.1 μA. (b) Schematic of the waveguide measurement setup. The sample is placed in a dilution refrigerator accessible via C-coated windows. (c) Microscope view of a device used for chip alignment.

For the waveguide measurements, the sample is loaded into a dilution refrigerator. Figure 2b shows a schematic of the experimental setup. A stack of four C-coated windows at room temperature, 40, 3, and 0.100 K at the base of the refrigerator permits optical access to the device under test. The critical temperature of our superconducting film is *T*_c_ ∼ 12 K: a similar performance could be obtained at a significantly higher temperature,^23^ without using a dilution refrigerator. A lens (Thorlabs C660TME-C) objective with numerical aperture 0.6 (*f* = 2.97 mm) focuses the optical signal on the grating coupler designed for vertical coupling and optimized to preferentially couple the TE_00_ mode of the optical waveguide. Inside the cryostat, the position of the chip is adjusted with a three-axes slip-stick piezo stack (Attocube). The polarization of the incoming photons can be adjusted, and the coupled input power is measured.

The laser is focused onto the input grating of specific devices designed to optimize the coupling procedure (Figure 2c). These devices have both the input and the output structures within the field of view of the focusing lens, and the output grating is monitored for coarse adjustments. After that, a finer alignment on the measured devices was obtained by monitoring the count rate of the SNSPD to maximize the coupling. Limitations in the free space measurement setup prevented a direct measurement of the grating coupling efficiency. From simulations considering the specific excitation settings in the cryostat and systematic experimental characterization of the waveguide coupling and propagation losses performed at room temperature (see also the Supporting Information), the cryogenic coupling efficiency was estimated to be ∼2% at λ ∼ 1550 nm (see Figure 4b, inset). Accordingly, we estimate an overall system efficiency of 10^–8^ including grating couplers and circuit losses in cryogenic experiments. Figure 3a presents a simulation of the excited optical mode at 1550 nm. The NbTiN hairpin detector is visible on top of the ridge waveguide. Based on this mode distribution, the simulated absorption efficiency of an SNSPD versus its length is shown in Figure 3b. We chose an absorption length of 150 μm (corresponding to a hairpin detector with total length of 300 μm) to obtain a theoretical absorption efficiency >99.9%, robust against small misalignments between the two lithographic steps, as discussed in the Supporting Information. The photon count rate (PCR) for a device measured at a wavelength of 1550 nm and the corresponding dark counts are shown in Figure 3c as a function of the bias current. The critical current of this device is *I*_C_ ∼ 12.5 μA. The device has a low dark count rate, and in the PCR an indication of a plateau, suggesting good internal detection efficiency, can be seen. The timing jitter of the SNSPDs is measured through the waveguide and is shown in Figure 3d. The fit of an exponentially modified Gaussian leads to a FWHM of 93.3 ps. The measurement, at a bias current of 11.8 μA, uses a pulsed picosecond laser (APE PicoEmerald, 3 ps pulse, 80 MHz repetition rate) and a high-speed oscilloscope by LeCroy (WaveRunner, 640Zi, frequency: 4 GHz, 40GS/s). The jitter could be improved by microwave engineering the contact leads (Figure 1c) and cryogenic low noise amplification.

**Figure 3:**
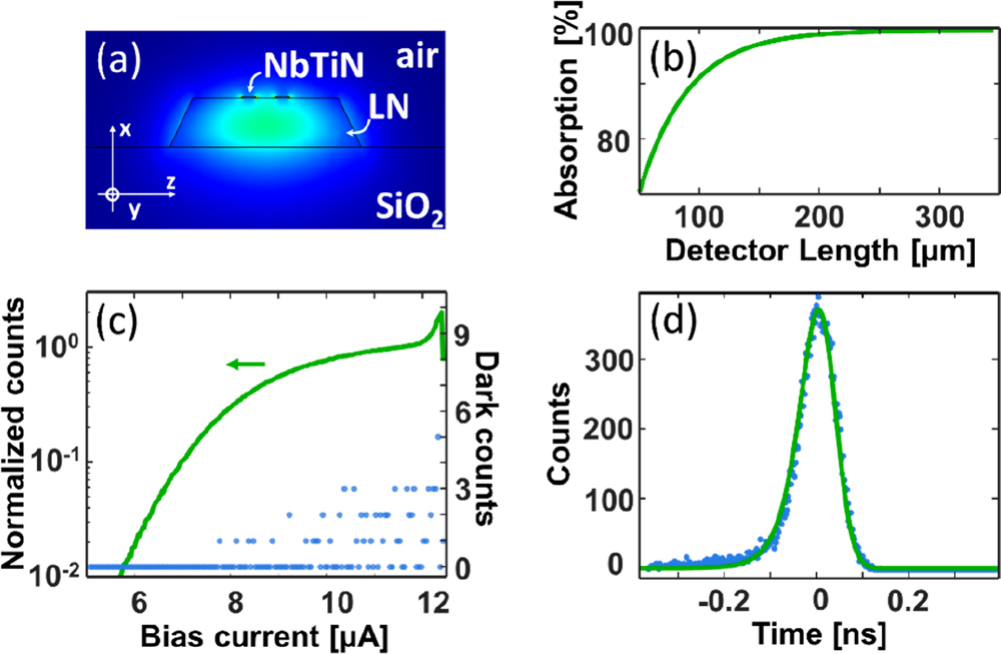
(a) Optical mode in a 300 nm thick and 700 nm wide TFLN ridge waveguide, with 65° sidewall angle. On top of the waveguide are the two stripes of the hairpin SNSPD. (b) Simulated detector absorption as a function of its total length for a signal at 1550 nm. (c) Guided-wave photon (green line) and dark (blue dots) counts measured at 1550 nm as a function of *I*_bias_. (d) Jitter of the device: experiment (blue dots) and fit (green line).

**Figure 4:**
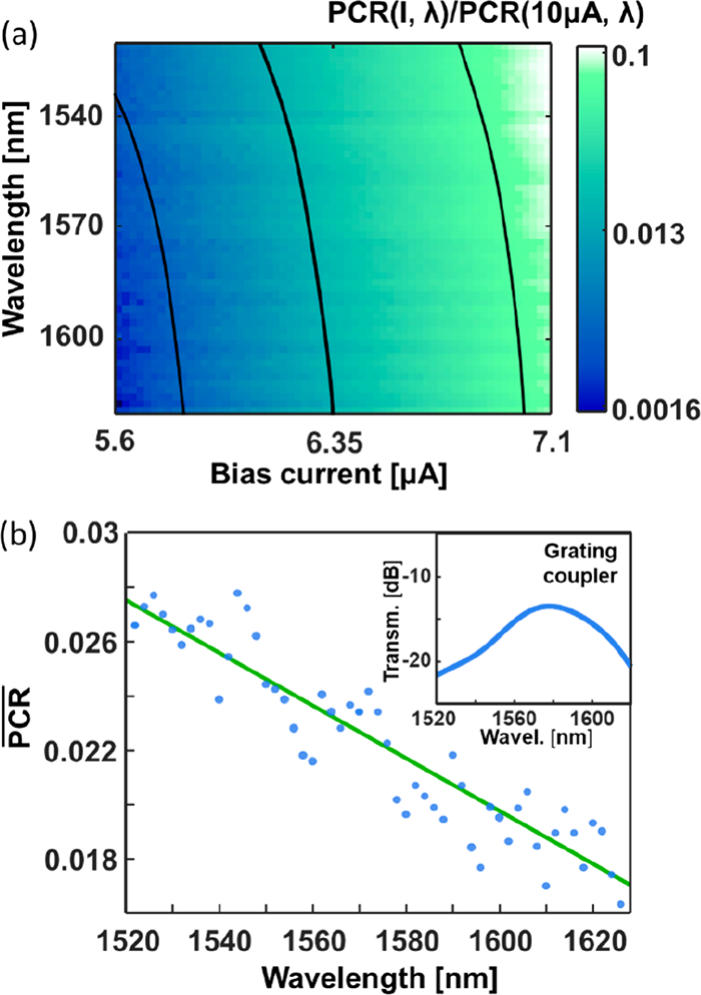
(a) Photon count rate (PCR) map showing the normalized responsivity of the hairpin detector for different wavelengths and bias currents. Averaged contour lines are plotted to highlight the trend in the measurement. (b)  calculated with the formula described in the main text: experimental data (blue dots) and linear fit (green line). Inset: simulated coupling efficiency of the grating couplers. The simulation is based on atomic force microscopy of the fabricated grating couplers.

The PCR for a waveguide coupled SNSPD is wavelength dependent, as highlighted by Figure 4a, mapping the PCR normalized to its value at 10 μA as a function of the bias current and optical wavelength. To analyze the wavelength dependence of the detector and remove artifacts from the optical circuit, such as standing waves or high-frequency variations in the grating coupler, we define the normalized average photon count rate ():
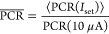
where ⟨PCR(*I*_set_)⟩ is the average PCR for a bias currents set point *I*_set_ between 5.6 and 7.1 μA, i.e., well below the saturation regime (occurring at *I* ≥ 10 μA where the change of the PCR as a function of the bias current *I* is limited). Thus, the  highlights the detection dynamics taking place at lower bias currents, where the total detection probability is lower (given the lower count rate), but the dependence of the detection event on the photon energy (wavelength) is most pronounced. The  as a function of λ is presented in Figure 4b. Its linear trend is not related to the coupling efficiency spectral dependence (simulated in the inset of Figure 4b), but to the intrinsic response of the superconducting detectors.^25^ For this relatively narrow wavelength range, a linear fit allows the wavelength to be extracted from the measurement. With a sensitivity of −9.7 × 10^–5^ nm^–1^ and a standard variation of the measurement samples of σ ∼ 0.06 to the linear fit, a wavelength accuracy in the order of 15 nm is obtained (Δλ = ±7.5 nm).

The result of Figure 4b is found by starting from the map of the normalized PCR for currents between 5.6 and 7.1 μA (plotted in Figure 4a), which represents a subset of the whole responsivity matrix, measured for currents between 0 and 15 μA. A better accuracy can be obtained choosing different bias currents set point based on the wavelength region of interest. Figure 5 reports the analysis for four different wavelength ranges (for λ = [1520, 1536] nm; [1557, 1571] nm; λ = [1580, 1594] nm; for λ = [1616, 1630]) for which the method described above gives an uncertainty below Δλ = ±4 nm when the bias current set point is properly chosen. The best result is obtained for the wavelength range 1616–1630 nm when *I*_set_ = [5, 5.3] μA. In this case, as visible in Figure 5d, the uncertainty is Δλ = ±2 nm. The correct choice of *I*_set_ permits us to obtain an uncertainty of ±4 nm over the whole C- and L-band (see also the Supporting Information). A very low power signal (to the limit of single photons) is enough to obtain this spectral resolution. We envision this functionality as a powerful resource in future wavelength-multiplexed quantum communication transceivers.

**Figure 5:**
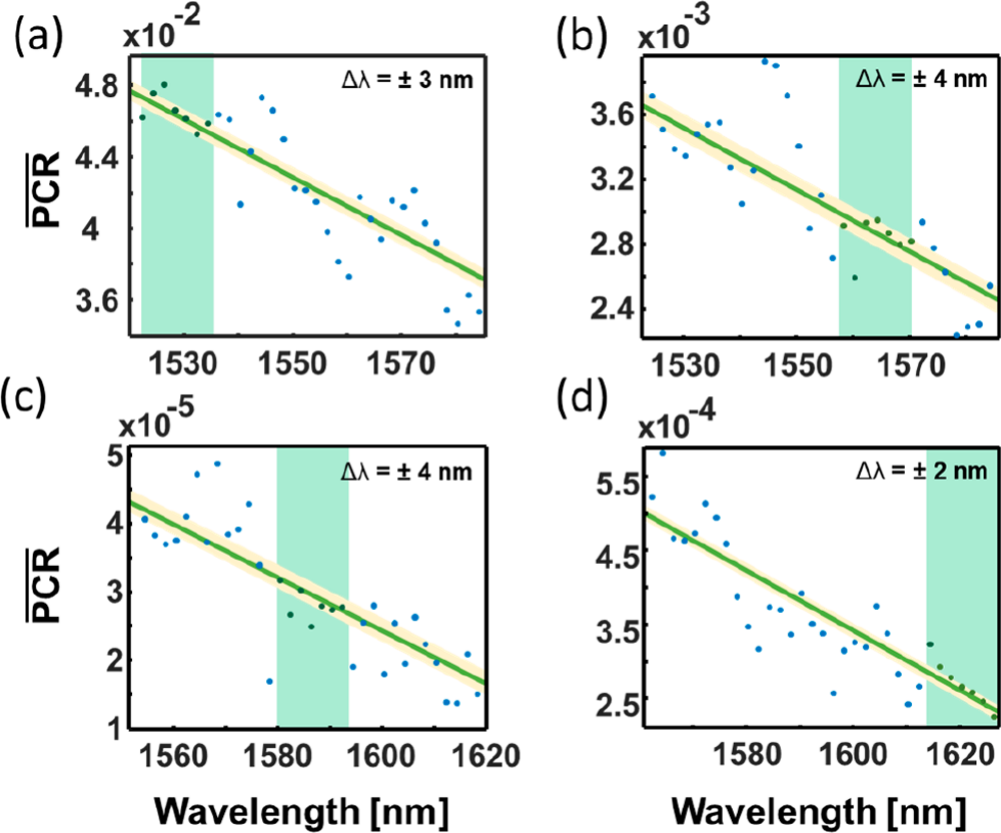
obtained for different bias current set points: experimental data (dots) and linear fit (green line). The yellow band represents the  range corresponding to an uncertainty of Δλ (±1σ) specified in each plot. The wavelength ranges of interest are highlighted in green and are (a) [1520, 1536] nm; (b) [1557, 1571] nm; (c) [1580, 1594] nm; and (d) [1616, 1630] nm. The bias current set points for the four cases are (a) *I*_set_ = [6.72, 6.85] μA; (b) *I*_set_ = [5, 5.3] μA; (c) *I*_set_ = [4.4, 4.52] μA; and (d) *I*_set_ = [5, 5.3] μA.

The measurements performed using a tunable laser (Toptica CTL) showed that at multiple bias points SNSPDs can be used as waveguide integrated wavelength meters. This in situ wavelength monitoring capability lends itself to implementation in many different photonic platforms. It is particularly appealing for its compactness and simplicity with respect to alternative solutions previously proposed for similar purposes in silicon photonics and relying on waveguide gratings^26,27^ or on multimode interferometer couplers.^28^ These alternatives, which split the incoming radiation into different optical waveguides based on their spectral components, show a higher complexity at the level of the optical circuit and a large footprint compared with ours.

To conclude, we report on SNSPDs integrated on TFLN optical waveguides. Leveraging the dependence of the detector response on the wavelength of the impinging photons, we implement an on-chip wavelength meter for the telecom C- and L-bands. Our device holds promise for the integration of all the building blocks needed for on-chip quantum optics on lithium niobate and the possibility of implementing new complex optical functions, such as wavelength detection, fully on-chip. This result further highlights the versatility of SNSPDs beyond quantum optics, leveraging their intrinsic device characteristics for grating-free spectroscopy integrated on the rapidly developing photonic platform of TFLN.

## Supporting Information Available

(1) Coupling efficiency of the TFLN waveguides; (2) accuracy of the waveguide integrated wavelength meter; (3) stability of the measurement setup; (4) robustness against misalignment of the SNSPD with respect to the waveguide (PDF)

## Notes

Parts of this work have been presented in the form of oral contribution at the CLEO-Europe conference in June 2023 (paper no. CK-1.2).
