# Supplementary material for: Wavelength-Sensitive Superconducting Single-Photon Detectors on Thin Film Lithium Niobate Waveguides
Source: Nano Lett. 2023 Oct 23;23(21):9748–52. doi: 10.1021/acs.nanolett.3c02324 (PMC10636877; doi:10.1021/acs.nanolett.3c02324)
Supplement: Supplementary file 1 — nl3c02324_si_001.pdf [file nl3c02324_si_001.pdf]

Wavelength sensitive superconducting single photon detectors on thin film lithium niobate waveguides

- *Supporting Information* -

Alessandro Prencipe [*,‡], *Samuel Gyger* [†,‡], *Mohammad Amin Baghban, Julien Zichi, Katharina D. Zeuner, Thomas Lettner, Lucas Schweickert, Stephan Steinhauer, Ali W. Elshaari, Katia Gallo, and Val Zwiller*

Department of Applied Physics, KTH Royal Institute of Technology, Roslagstullsbacken 21, Stockholm, SE-106 91, Sweden

* Corresponding author: Alessandro Prencipe: prenc@kth.se

‡ These authors contributed equally.

S-1. Coupling efficiency of the TFLN waveguides ***p. S2***

S-2. Accuracy of the waveguide integrated wavelength meter ***p. S3***

S-3. Stability of the measurement set up ***p. S5***

S-4. Robustness against misalignment of the SNSPD with respect to the waveguide ***p. S5***

S-1. Coupling efficiency of the TFLN waveguides.

A room temperature characterization was carried out to evaluate the response of dedicated test structures used to assess optical losses of waveguides without SNSPD. This allowed the systematic characterization (at T_{Room}) of grating couplers similar to the ones used for the devices discussed in the main text. Figure S-1 shows the measured transmission of one of these test structures (and the simulated response accounting for the effect of the grating couplers). The T_{Room} characterization was implemented using single mode fibers positioned in the proximity of the chip surface (instead of the focusing lens mounted in the cryostat described in the main text).

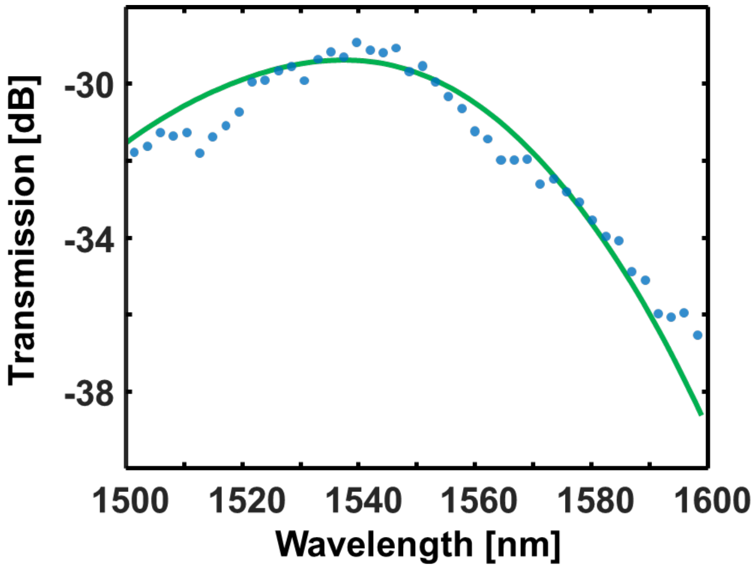

Figure S-1. Transmission spectrum obtained at room temperature from a waveguide structure used to characterize the optical losses and the grating coupler response. The plot shows both the experiment (blue dots) and the simulation (green line).

After replacing the fiber for a properly positioned lens and adjusting the geometrical parameters of the grating couplers (relying on the result of atomic force microscopy of the fabricated

devices), the same simulation tools was used to evaluate the response of the cryogenic device. The result of the simulation for the cryogenically characterized devices is shown in the inset of Figure 4(a) in the main text.

S-2. Accuracy of the waveguide integrated wavelength meter.

The complete responsivity matrix of the waveguide integrated wavelength meter accounts for wavelengths in the range: 1520 nm – 1630 nm and currents from 0 μA to 15 μA. By choosing bias currents setpoints between 5.6 and 7.1 μA (i.e. restricting the study to the subset of the responsivity matrix highlighted in Figure 4(a) in the main text), the normalized average photon count rate $\overline{PCR}$ provides a wavelength accuracy of ~ 15 nm on the whole C- and L-bands (see Fig. 4(b) in the main text). After this first rough characterization, the measurement accuracy can be improved selecting a different set of currents (I_{set}'). This is shown in Fig. 5 of the main text for four different wavelength ranges. Table S-T1 describes how to choose I_{set}' to obtain the optimal sensitivity for different wavelength ranges between 1520 nm and 1630 nm. Unless differently specified, the normalization current amounts to $I_{norm} = 10$ μA.

R1 ($\Delta\lambda \sim 7.5nm$) ($I_{set} = [5.6 - 7.1]\ \mu A$)	*Second iteration:* I_{set}' *[μA]*	*New Δλ [nm] (1σ)*	*R2*	*Third iteration:* I_{set}'' *[μA]*	*New Δλ [nm] (1σ)*
1520 < λ < 1536	$[6.9 - 7]$	$\sim 3\ nm$			
1536 < λ < 1552	$*[4.45 - 4.58]$	**$\sim 6\ nm$**	1536 < λ < 1544	$*[5.68 - 6.1]$	$\sim 3\ nm$
			1544 < λ < 1544	$*[5.68 - 6.1]$	$\sim 3\ nm$
1552 < λ < 1568	$[4.85 - 4.98]$	$\sim 4\ nm$			
1568 < λ < 1584	$[4.35 - 4.73]$	**$\sim 5.5\ nm$**	1568 < λ < 1576	$*[4.38 - 4.68]$	$\sim 3\ nm$
			1576 < λ < 1584	$[4.65 - 4.75]$	$\sim 3\ nm$
1584 < λ < 1600	$[5.2 - 5.38]$	$\sim 3.5\ nm$			
1600 < λ < 1616	$[3.9 - 7]$	$\sim 2.5\ nm$			
1616 < λ < 1628	$[5 - 5.35]$	$\sim 2\ nm$			

Table S-T1: Waveguide integrated wavelength meter improved accuracy ($*I_{norm}$=10.48 μA).

In the first column, R1 represents "range 1", a rough wavelength range obtained through the $\overline{PCR}$ measurement as per Figure 4(b) in the main text. Iterating a second time the analysis with different bias currents setpoints (I_{set}', in the second column of the table), it is possible to reduce the wavelength uncertainty ($\Delta\lambda$, see the third column of the table). In some cases, it is worth repeating the process a third time (starting from the information acquired in the second iteration, "range 2" - R2) to further reduce $\Delta\lambda$. Here, as in the main text, the uncertainty $\Delta\lambda$ is defined as one standard deviation of the experimental points from the linear fit.

Different devices were probed (see also Figure S-2). The best accuracy we could measure amounts to ~ 4 nm, corresponding to an uncertainty of $\pm$ 2 nm, for the case represented in Fig. 5(d) in the main text. An uncertainty of $\pm$4 nm was obtained on the whole C- and L-bands. Our measurements are limited by coupling efficiency and electronic noise. Improved results could be obtained with better coupling structures and cryogenic amplification of the detector's output.

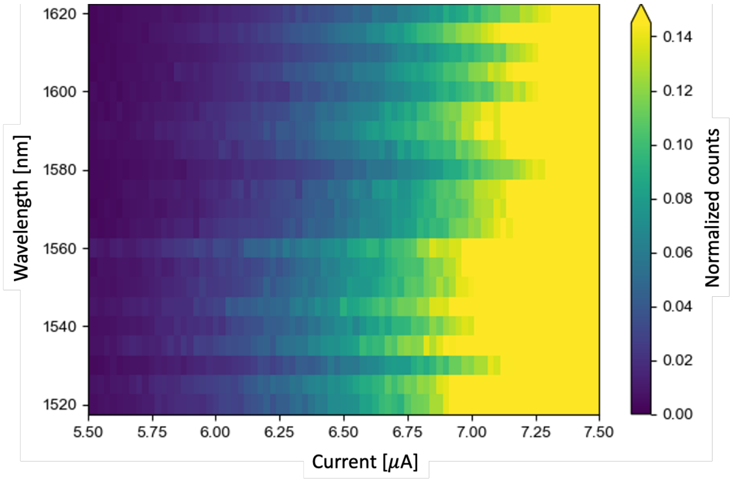

Figure S-2. PCR map for a different device from the one analyzed in the main text. The wavelength meter performances are similar.

All the measurements we report on the wavelength meter capabilities were performed using CW laser sources. The wavelength meter performance would be negatively affected by waveguide dispersion if short light pulses were used as optical input. Dispersion free waveguides for the wavelength range of interest could be engineered to retrieve a good wavelength meter performance also in case of short light pulses signals.

S-3. Stability of the measurement set up.

The robustness of our measurements against vibrations and alignment drifts is granted by choosing a proper integration time and by mounting the focusing lens (used to couple the optical signal into the waveguides) monolithically with respect to the chip. In this way the environmental disturbances leading to a small perturbation of the path followed by the input beam correspond to a negligible change in the position where the input is collimated onto the grating coupler. This was tested (for multiple wavelengths) by monitoring the number of counts

while inputting constant optical power and keeping fixed the bias current. The result of this analysis is plotted in Figure S-3.

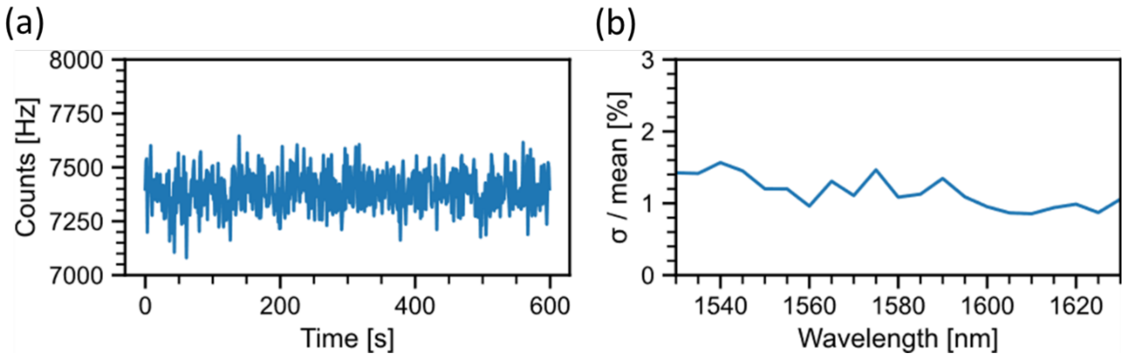

Figure S-3: Stability of the measurement. (a) Count rate of a device measured over 10 minutes at a wavelength of 1550 nm. b) Normalized standard deviation of the counts for multiple wavelengths over a total measurement time of 3.5 hours.

The data above (Fig. S-3(a)) were collected at a frequency of 1 Hz (same or lower than the one used to obtain the photon count rates presented in the main text), which makes the system insensitive to vibrations. The number of counts remains stable over time and its variation stays below 1.6% for different wavelengths (Fig. S-3 (b)), confirming the stability of our measurement setup.

S-4. Robustness against misalignment of the SNSPD with respect to the waveguide.

As described in the main text (Fig. 1(a)) the waveguide integrated SNSPDs are obtained relying on two electron beam lithographic steps. Electron beam lithography typically leads to very small misalignment errors. Still an alignment inaccuracy in the order of ~100 nm between the two steps may occur. Figure S-4 shows that long enough detectors are robust against such

misalignment. Figure S-4(b) compares the simulated absorption efficiency for a perfectly centered device (already shown in Fig 3(b) in the main text) with the one of a SNSPD 130 nm skewed from the middle of the optical waveguide (Fig S-4(a)). With a total detector length of 300 μm (like the ones studied in our experiments) we get:

Absorption efficiency of the centered SNSPD = 99.94%, comparable with

Absorption efficiency of the non-centered SNSPD = 99.87%.

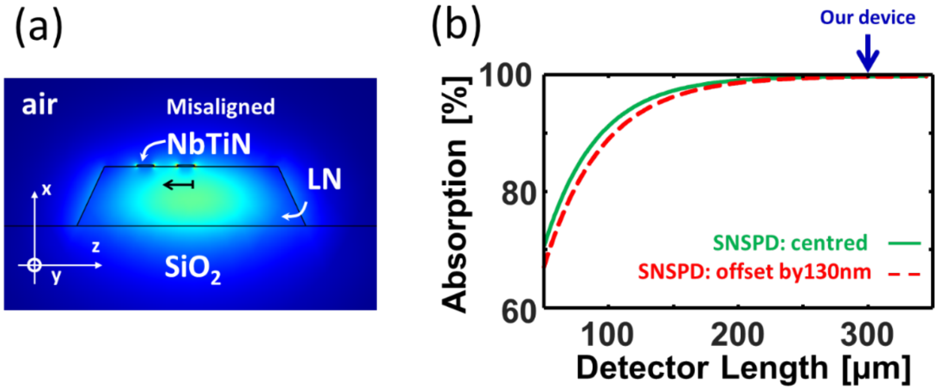

Figure S-4: (a) Mode simulation accounting for a misaligned detector (waveguide thickness = 300 nm, waveguide width = 700 nm). (b) Absorption efficiency for both a perfectly centered detector (solid green line, reproduction of Fig. 3(b) from the main text) and for a detector offset by 130 nm with respect to the center of the $LiNbO_3$ nanowaveguide (broken red line). A total detector length of 300 μm makes the device robust against the misalignment.
